# Prevalence of Burnout Among Healthcare Workers During the COVID-19 Pandemic: A Systematic Literature Review

**DOI:** 10.1192/bjo.2025.10204

**Published:** 2025-06-20

**Authors:** Beebee Zeba Mahetaab Mubarak Jan, Sumera Bibi Keenoo

**Affiliations:** 1 King’s College University, London, United Kingdom; 2 University of Mauritius, Reduit, Mauritius

## Abstract

**Aims:** Burnout among healthcare workers has been a significant issue exacerbated by the COVID-19 pandemic. This review aims to synthesise the existing literature on the prevalence, signs, symptoms, and risk factors of burnout among healthcare workers during the pandemic.

**Methods:** This systematic review follows the PRISMA guidelines. We searched the Web of Science and Scopus for relevant studies published between January 2020 and December 2022. Inclusion criteria were studies reporting burnout prevalence among healthcare workers during the COVID-19 pandemic. Data were extracted and analysed using a structured framework

**Results:** The review included 50 studies, with a total sample size of 30,000 healthcare workers. Prevalence of burnout varied significantly across regions, with the highest rates reported in Saudi Arabia (75%) and Kuwait (76.9%). Common symptoms included emotional exhaustion, depersonalisation, and reduced personal accomplishment. Key risk factors identified were high work demands, lack of personal protective equipment (PPE), and prolonged working hours.

**Conclusion:** The COVID-19 pandemic has significantly impacted the mental health of healthcare workers, leading to high burnout rates. Tailored interventions are needed to address this issue and support healthcare workers during global health emergencies.

